# Employee Competitive Attitude and Competitive Behavior Promote Job-Crafting and Performance: A Two-Component Dynamic Model

**DOI:** 10.3389/fpsyg.2018.02223

**Published:** 2018-11-21

**Authors:** Haifeng Wang, Lei Wang, Chunquan Liu

**Affiliations:** ^1^Sunshine Life Insurance, Beijing, China; ^2^School of Psychological and Cognitive Sciences, Beijing Key Laboratory of Behavior and Mental Health, Peking University, Beijing, China

**Keywords:** competition, competitive attitude, competitive behavior, trait competitiveness, sales performance, team competitive climate, job crafting

## Abstract

While competition has become increasingly fierce in organizations and in the broader market, the research on competition at an individual level is limited. Most existing research focuses on trait competitiveness. We argue that employee competitiveness can be state-like and can be demonstrated as an attitude toward and behavior representative of competition. We therefore propose a dynamic model with two separate components: *competitive attitude* and *competitive behavior*. Drawing upon self-determination theory and the person–environment interaction perspective, we examine how employee competitive attitude and competitive behavior can be influenced by both personal characteristics and team climate, which in turn leads to different work outcomes, as demonstrated in two studies. Study 1 developed measures for competitive attitude and competitive behavior. Study 2 collected data from salespeople in a large insurance company in three waves. The results showed that employee competitive attitude and behavior could be predicted by personality. Moreover, employee competitive attitude and behavior were related to sales performance in differential ways via job crafting, and these mediated relationships could be moderated by team climate. These findings support the two-component dynamic model combining competitive attitude and behavior, which helps promote understanding of the dynamics of competition and its consequences at the individual level. Theoretical and practical implications are also discussed.

## Introduction

Competition and/or competitive advantage have been considered the most important survival method for individuals, organizations, and society (Thiel, 2017). It is even viewed as a component of human nature (Churchill et al., 1997; Klein and Miller, 1998; Matsumoto and Willingham, 2006; Dohmen et al., 2011), and human beings develop through competition with the environment and within the species (Kohn, 1992; Carroll and Tomas, 1995; Pfeffer and Sutton, 2000; Birkinshaw, 2001; Marino and Zábojník, 2004; De Waal-Andrews and Van Beest, 2018). Given its importance, scholars from many areas, including economics (e.g., Klemperer, 1995; Porter, 1998; Fehr and Schmidt, 1999; Aghion et al., 2005), sociology (Hayward and Kemmelmeier, 2007), politics (e.g., Martimort and Semenov, 2008; Besley et al., 2010; Becker-Ritterspach and Dörrenbächer, 2011), management (e.g., Hum and Sim, 1996; Ketchen et al., 2004; Blanes and Nossol, 2011; Kuhlen and Tymula, 2012), and psychology (for recent studies, see Wittchen et al., 2013; De Waal-Andrews and Van Beest, 2018), have paid much attention to competition. While research in other domains usually focuses on macrolevel analysis, psychology primarily engages in microlevel analysis. In particular, psychologists usually examine competition as special personality trait (Spence and Helmreich, 1983).

However, competition in human beings is more complicated than we think. People high in trait competition may not have to demonstrate competitive behavior, while those low in trait competition may have to show competitive behaviors. The studies examining competitive behavior and its mechanisms and marginal conditions at the micro level are still lacking. We know little about how trait competition, competitive attitude, and competitive behavior relate to each other, and there have been no measures estimating competitive attitude and competitive behavior. This study will address these issues.

In particular, we propose a dual-dynamic model of competitive attitude and competitive behavior and examine how competitive attitude and behavior relate to trait competition as an individual factor and competitive climate as an environmental factor in an effort to discover how their interactions might influence the performance in workplace. We will first review the literature and then propose our research model and hypotheses.

### Competition and Its Function

Although competition is a very common phenomenon, its definition is not consistent. There are two main perspectives: one focuses on limited resources, and the other focuses on social status. According to the perspective of limited resources, competition is framed as a concept that describes a situation where individuals or organizations via for limited resources or rewards (Kohn, 1992). In this way, competition is defined as the separate pursuit of the same scarce resources (McPherson, 1983; Burt, 1992; Bothner et al., 2007; Ingram and Yue, 2008). Competition is believed to be very essential to the efficient allocation of scarce resources and to be very important to promoting creativity and innovation (Carroll and Tomas, 1995; Birkinshaw, 2001; Marino and Zábojník, 2004). It can involve either depletion of the same resource at different times, with no direct interactions between agents (scramble competition) or with direct aggressive confrontation between competing agents (e.g., Isbell, 1991; Henson and Cushing, 1996). Consequently, those who do better or who are good at competition may perform better.

Another perspective on competition is based on social comparison. People have an innate drive to be the best. They take horizontal social comparison and try their best to transcend others. Researchers claim that people enjoy prevailing over others (Klein and Miller, 1998; Matsumoto and Willingham, 2006; Dohmen et al., 2011). This is particularly because outperforming others can make people feel proud (Tesser and Collins, 1988; Exline and Lobel, 2001) and successful (Thompson et al., 1995).

Actually, both perspectives view competition as a means to outperform others and survive effectively. This leads to a link between competition and performance and, eventually, a belief that people with highly competitive traits can be more successful.

The relationship between competition and performance is based on the logic of zero-sum contests in which two or more agencies go head-to-head, and one wins at the expense of the other (Deutsch, 1949). According to this logic, people outperform others via competition. To investigate this relationship, psychology employs microanalysis to account for and consider individual and/or group characteristics that may link competition and performance (e.g., Wittchen et al., 2013).

### Competitive Traits and Performance

Trait competitiveness is a kind of personality that refers to “the enjoyment of interpersonal competition and the desire to win and be better than others” (Spence and Helmreich, 1983, p. 41). Kohn (1992) proposed a concept called “intentional competitiveness” that depicts an internal desire to be the best. It describes someone who enjoys competing with and surpassing others. These two concepts are generally thought to be similar or consistent (Brown et al., 1998), both focusing on the individual’s internal characteristics that drive a person to outperform others.

However, the previous research findings about the relationship between trait competitiveness and performance are mixed. While some research found that trait competitiveness was positively correlated with individual and company performance (e.g., Carsrud and Olm, 1986; Brown and Peterson, 1994), other research did not find a significant relationship, and some even found a negative relationship between trait competitiveness and performance (Spence and Helmreich, 1983). This contradiction suggests that there are some other factors that may play important roles in affecting the relationship between trait competitiveness and performance. Trait competitiveness is only one intra-individual factor that may relate to performance; whether it can lead to high performance depends on other internal and external factors.

Some researchers have identified some external, contextual factors such as competitive climate (Brown et al., 1998) that moderated the relationship between trait competitiveness and performance. Drawing upon self-determination theory (Deci, 1980; Ryan and Deci, 2000, 2006, 2017), we argue that some intrapersonal factors, such as attitudes and behavior toward others, as proximal variables in a competitive context may also influence the performance and/or the relationship between trait competitiveness and performance. Self-determination theory claims that some intrinsic motives can play important roles in individual’s behavior (Deci, 1980). It is possible that trait competitiveness as a static-like factor drives personal attitude and behavior in the very beginning. Trait competitiveness can be an antecedent variable of competitive attitude and competitive behavior that finally results in performance output.

*H1*: Trait competitiveness is an antecedent factor of competitive attitude and competitive behavior.

### Competitive Attitude and Behavior, Job Crafting, and Job Performance

In this study, we propose two concepts, competitive attitude and competitive behavior, both of which function as proximal factors that relate directly to performance. Drawing upon the basic concept of attitude (Eagly and Chainken, 1993), we define *competitive attitude* as a belief concerning whether an individual likes competition. In addition, we define *competitive behavior* as the actual actions people take or are inclined to take in a specific job or life environment to compete for resources or succeed over others. These two concepts are related to competition but are distinct from trait competitiveness. While trait competitiveness refers more to a static individual disposition, competitive attitude, and behavior involve a dynamic psychological status: they are changeable, cultivatable, and can vary across different contexts (Cialdini et al., 1981).

Many workplace attitudes have been found to be correlated to job performance, such as organizational commitment (Meyer and Herscovitch, 2001) and commitment to change (Chen and Wang, 2011). Similarly, we believe that competitive attitude, as a kind of workplace attitude, also contributes to job performance. Those high in competitive attitude devote more effort and energy, which in turn leads to high performance.

Distinct from trait competitiveness but akin to competitive attitude, competitive behavior is similarly dynamic, and can be exhibited even without trait competitiveness. That is, people low in trait competitiveness can also exhibit competitive behavior in certain circumstances, particularly in a competitive climate. However, according to the perspective of social psychology that behavior and attitude are substantially different (Wicker, 1969; Kraus, 1995), competitive behavior can also be different from competitive attitude. Consider the following situation. A person may not like competition and therefore harbors a negative attitude toward competition. However, that person may still demonstrate competitive behavior under some situations, particularly in a highly competitive climate. Driven by the organizational competition environment, the person has to compete with others to survive. In this manner, competitive behavior is highly dynamic, depending heavily on the situation.

We believe competitive behavior is closely related to job performance since it is more closely related to job behavior and performance. People showing more competitive behavior tend to outperform others and are more likely to do their best at work, thereby potentially resulting in better job performance.

### The Mediating Role of Job Crafting in the Relationship of Competitive Attitude and Behavior With Job Performance

The present research also examines the mechanism that governs how competitive attitude and competitive behavior relate to job performance. In particular, we argue that those high in competitive attitude and competitive behavior would show more job crafting behavior (Tims et al., 2012); that is, they will try their best to seek resources for their work and get extra information and support for their job (Wrzesniewski and Dutton, 2001), such that they would achieve a higher level of job performance.

Job crafting is a process wherein employees redesign their jobs by actively selecting tasks, adjusting job contents, and making their job more meaningful (Wrzesniewski and Dutton, 2001; Berg et al., 2008; Parker and Ohly, 2008). It has been found that job crafting can facilitate job satisfaction and promote fast job adjustment and high-speed development (Parker and Ohly, 2008). It can also improve the person-job fit. Employees high in job crafting also rapidly adapt to job demands and resources (Tims and Bakker, 2010; Tims et al., 2012), and by doing so, they can get more influence and valuable feedback at work, which helps increase their work performance and thereby better realize their career goals (Berg et al., 2010).

**FIGURE 1:**
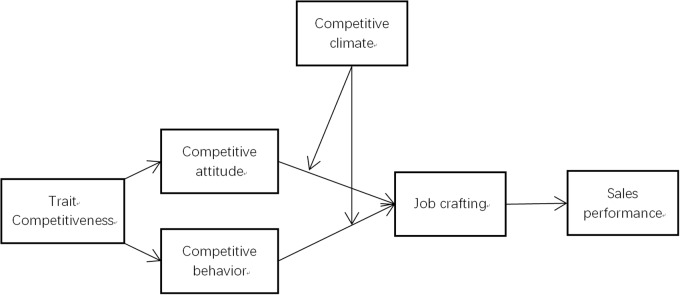
Research model.

Taken together, we think that both competitive attitude and competitive behavior can drive more job crafting behavior that, in turn, helps achieve better job performance. Here, we propose a two-component dynamic model linking two changeable, dynamic variables, competitive attitude, and competitive behavior, to performance. They are two proximal variables, closely related to job behavior and performance. Both can be traced back to trait competitiveness. However, they are not trait-like or static. Instead, they are more situation-driven, more adjustable, and can be shaped by the environment to be suitable to the job and/or situation requirements (see Figure 1). Thus, we propose that:

*H2a*: There is a congruent effect of competitive attitude and behavior on job crafting. Competitive attitude and behavior are more positively related to job crafting at higher congruence.
*H2b*: There is a congruent effect of competitive attitude and behavior on job performance. Competitive attitude and behavior are more positively related to job performance at higher congruence.
*H2c*: Job crafting is higher when the levels of competitive attitude and behavior are high.
*H2d*: Job performance is higher when the levels of competitive attitude and behavior are high.
*H3a*: Competitive attitude leads to better job performance via job crafting; employees with a high level of competitive attitude will demonstrate more job crafting behavior that in turn will help them achieve a higher level of performance.
*H3b*: Competitive behavior leads to better job performance via job crafting; employees with a high level of competitive behavior will demonstrate more job crafting behavior that in turn will help them obtain a higher level of performance.

### Competitive Climate and Competitive Behavior

The competitive psychological climate was originally proposed by Kohn (1992). It is defined as the extent to which employees feel their reward is determined according to the comparison of their performance with others. The so-called competitive climate is a kind of organizational climate under which employees have to compare their performance with others, which leads to a competitive feeling and pressure. This climate drives employees to be competitive, no matter whether the employees themselves are trait-like competitive.

Unfortunately, the existing findings on the influence of competitive climate and its interaction with trait competitiveness on performance are mixed. Beersma et al. (2003) proposed that a competitive work environment would make people go to any length in their efforts to be better than others. They showed that a competitive situation would influence the performance of a team engaged in a low-interdependence task. Brown et al. (1998) found that a competitive climate would interact with personal competitive traits of salespersons to influence goal setting and performance. However, Fletcher et al. (2008) examined the influence of competition as an interaction between trait competitiveness and competitive climate and found that competitive climate could negatively affect supervisor-rated task performance for those high in trait competitiveness. Specifically, supervisors rated employees with high levels of trait competitiveness lower in performance when the team was perceived as being more competitive. We argue that this finding may be due to their sample consisting of information technology employees accustomed to achieving better performance due to their tendency to work with a highly interdependent team. The result can be very different for individuals working as part of a team on a low-interdependent task that may require and allow more competition, according to Beersma et al. (2003), such as a sales team in the insurance industry.

On the other hand, as we mentioned above, competitive attitude and behavior are more proximal to performance (via job crafting). Moreover, according to the person–environment (P–E) fit perspective, an individual’s personal characteristics and environmental factors interact to influence individual’s attitude and behavior (Endler and Magnusson, 1976; Pervin, 1989; Edwards, 1991; Kristof, 1996; Kristof-Brown et al., 2005). We further the existing literature to argue that it can be possible that the competitive climate interacts with competitive attitude and competitive behavior to influence job crafting, thereby affecting job performance, the final outcome variables. Specifically, when the competitive climate is weak, the employee with high competitive attitude and competitive behavior, who shows that he wants to be best in workplace, will be isolated by others. And isolation has a negative effect on output such as job crafting and performance (Mulki et al., 2008). On the contrary, when competitive climate is strong, performance will not be affected without isolation. The competitive climate sets up an environment where employees would be more likely to compare and compete with peers (Kohn, 1992). It can be an important environmental supplementary factor to performance particularly when individual factors (like competitive attitude and behavior) are lacking. That is, a strong competitive climate can be a situational force that drives employees to show more job crafting and performance particularly when the level of competitive attitude and behavior is low. When the competitive climate is weak, the personal competitive attitude and behavior play more important roles in driving job crafting behavior, whereas when competitive climate is strong, this environmental power is efficient enough such that the relationship of personal competitive attitude and behavior with job crafting would not be very close. In other words, there is an *interactive effect* between competitive climate (the environmental factor) and competitive attitude/behavior (the personal factors). Thus, we propose the following hypotheses:

*H4a*: Competitive climate will interact with competitive attitude to influence personal job crafting such that, under the high level of competitive climate, the relationship between competitive attitude and job crafting will be stronger.
*H4b*: Competitive climate will interact with competitive behavior to influence personal job crafting such that, in a highly competitive climate, the relationship between competitive behavior and job crafting will be stronger.
*H5a*: Competitive climate will moderate the indirect effect in Hypothesis 3a such that it is stronger for individuals in a highly competitive climate (vs. those in a less competitive climate).
*H5b*: Competitive climate will moderate the indirect effect in Hypothesis 3b such that it is stronger for individuals in a highly competitive climate (vs. those in a less competitive climate).

Figure 1 shows the research model of the present research.

### Overview of the Present Research

We performed two studies to test our research model. Study 1 developed a measure of competitive attitude and competitive behavior and tested its reliability, structure, and concurrent validity. Study 2 tested the effect of competitive attitude and competitive behavior on performance and investigated the mediating effect of job crafting and the moderating effect of competitive climate using multiwave, multisource data. This research was in accordance with the Declaration of Helsinki and was approved by the Ethics Committee of School of Psychological and Cognitive Sciences at Peking University. For both studies, the data were collected online. In this case, the written informed consent was not obtained from each participant. Instead, we showed all participants their rights on a smartphone or computer screen before the start of the questionnaire and told them that if they truly understood their rights and wanted to participate in the study, they could push the button on the bottom of the screen to start. In this way, we obtained the participants’ electronic informed consent.

## Study 1: Measures Development

### Methods

#### Participants

Participants for exploratory factor analysis (EFA) were 155 employees who worked for an insurance company. Each employee received a link via smartphone and was asked to fill out the questionnaire on their phone. Their average age was 24.72 years old, 78 males (50.3%).

Participants for confirmative factor analysis (CFA) were recruited by snowballing online via smart phone. They first received a link. They were then informed their rights and asked if they wanted to participate in the survey in exchange for 1-dollar reward and feedback about their competitiveness. In total, there were 208 employees from a variety of industries such as IT, manufacturing, construction, finance and banking, education, and others, covering the spectrum of competition in different industries that participated in the study. Their average age was 35.06 years (*SD* = 5.04), 154 females (74%).

#### Measures and Procedures

##### Competitive attitude and behavior

Based on the definition and our main concern that focusing on the difference of attitude and behavior from trait competitiveness, we developed the measurement of competitive attitude and competitive behavior. Six graduate students and one professor in industrial and organizational psychology worked together to generate the original items and decide which item was suitable for each scale. An item was accepted for inclusion only when all group members agreed. Finally, each of two scales included five items (see Appendix for detail). Both scales were measured on a seven-point Likert scale, ranging from 1 (*totally disagree*) to 7 (*totally agree*).

##### Trait competitiveness

Trait competitiveness was measured using a scale developed by Helmreich et al. (1978). There are four items in the scale. A sample item is, “It is important to me to perform better than others on a task.” The scale was measured on a seven-point Likert scale, ranging from 1 (*totally disagree*) to 7 (*totally agree*).

##### Type A personality

Type A behavior pattern is used to describe those persons who are “aggressively involved in a chronic, incessant struggle to achieve more in less and less time, and if required to do so, against the opposing efforts of other things or other persons” (Friedman and Rosenman, 1974, p. 67). Type A personality has been found to be related to competition (see Price, 1983 for review). We thus included Type A personality as a validity criterion. Type A personality was usually measured using a scale developed by Friedman and Rosenman (1959). There are traditionally 60 items. To fit the online survey situation, we used the 8-item shortened version for the current study (Zhang, 1985). Two sample items are, “I feel I have the ability to do everything well,” and “People think I am a competitive person.” The participants replied either “yes” or “no” when asked whether the descriptor was applicable to them.

### Results and Discussion

Since we developed a new scale for both competitive attitude and competitive behavior based on our definition of each term, we first conducted the exploratory factor analysis (EFA) with all 10 items of competitive attitude and competitive behavior scales using SPSS 22.0. The results showed that the Kaiser–Meyer–Olkin measure of sampling adequacy was 0.85, indicating that the data were suitable for factor analysis. Parallel analysis indicated that two factors were significant and should be retained: Factor 1 had an eigenvalue of 4.66 (46.59% of the variance) and factor 2 had an eigenvalue of 2.70 (27.00% of the variance). As the good-of-fit test was significant (χ^2^ = 64.61, *p* < 0.00), and each item loaded onto the assumptive factor with a salient loading (loading > 0.30), no item should be removed. The results preliminarily confirmed the structure of competitive attitude and competitive behavior scales.

Then we tested the conceptual structure of the measurement by confirmative factor analysis (CFA). The analysis was performed using LISREL 8.7. The results (see Table 1) showed that the two-factor model fit the data well, with each item loaded onto the designated factor, χ^2^/df = 2.57, root mean square error of approximation = 0.087 (90% CI [0.065, 0.11]), comparative fit Index = 0.98, goodness of fit index = 0.92, and normed fit index = 0.96. All the results were statistically acceptable. These results suggested that the two scales had a good structural validity.

**Table 1:** CFA fitting index for competitive attitude and competitive behavior in Study 1.

Model	χ^2^/df	RMSEA (90% CI)	*P*	CFI	GFI	NFI
Two factors	2.57	(0.065, 0.11)	<0.001	0.98	0.92	0.96

*RMSEA, root mean square error of approximation; CFI, comparative fit index; GFI, goodness of fit index; NFI, normed fit index*.

To examine the concurrent validity of our two new scales, we collected the data for competitive attitude, competitive behavior, the trait competitiveness, and Type A personality. The means, standard deviations, correlations, and reliability estimates of all variables in Study 1 are shown in Table 2. The results showed that competitive attitude was significantly correlated with trait competitiveness, *r* = 0.41, *p* < 0.05; it was also significantly correlated with Type A personality, *r* = 0.20, *p* < 0.01. Similarly, competitive behavior was significantly correlated with trait competitiveness, *r* = 0.56, *p* < 0.01; it was also significantly correlated with Type A personality, *r* = 0.15, *p* < 0.05. Moreover, competitive behavior was moderately correlated with competitive attitude, *r* = 0.31, *p* < 0.01.

**Table 2:** Means, standard deviations, correlations, and reliability estimates in Study 1.

Variable	Mean	*SD*	1	2	3	4	5	6
(1) Gender	–	–	–					
(2) Age	35.06	6.34	0.02	–				
(3) Trait competitiveness	3.53	0.75	-0.17*	-0.02	(0.80)			
(4) Competitive attitude	3.62	0.86	-0.14*	0.16*	0.41**	(0.89)		
(5) Competitive behavior	3.53	0.87	-0.20**	0.03	0.56**	0.31**	(0.89)	
(6) Type A personality	5.73	1.89	-0.12	0.09	0.20**	0.20**	0.15*	(0.61)

*N = 208. SD, standard deviation. Cronbach’s alpha values are presented on the diagonal, and correlations are presented below the diagonal. Gender: 1 = male, 2 = female.*

*^∗^*p* < 0.05, ^∗∗^*p* < 0.01, two tailed*.

It can be seen that all observed variables were significantly correlated with each other, but the level of correlation was not very high, meaning that these variables are related to each other, but that they are not the same. We noted that the correlations between trait competitiveness and competitive attitude/behavior were moderate, suggesting that competitive attitude and behavior are dynamic variables that can be different from the trait competitiveness as a static-like variable. The alpha coefficients for all variables were between 0.61 and 0.89, all of which are acceptable. These results provided preliminary evidence for the reliability and validity of our two new scales and supported their usefulness in the subsequent study. The results also provided preliminary support for Hypothesis 1 that trait competitiveness can predict competitive attitude and competitive behavior.

## Study 2: Competitive Attitude and Behavior Predict Performance

### Method

#### Participants

Salespeople who worked for an insurance company and sold life insurance via telephone were recruited to be participants. We collaborated with the company to estimate its competitive climate. Each employee received a link via smart phone so that they could participate in the survey online. They were informed of their rights and the benefits of participation and that they could quit at any time. They were asked to fill out the questionnaire on their smartphone twice, with a 1-month interval between the response dates. We obtained a sample of 283 employees, 148 males (52.3%); their mean average age was 24.83 years (*SD* = 4.64). Their average job tenure was 12.53 months (*SD* = 15.22).

#### Measures and Procedures

##### Competitive attitude, competitive behavior, and trait competitiveness

These variables were measured in the same way as in Study 1.

##### Competitive climate

The competitive climate was measured by a scale developed by Brown et al. (1998). It is a four-item scale. A sample item is, “The amount of recognition you get in this company depends on how you perform compared to others.” The scale was measured on a seven-point Likert scale, ranging from 1 (*totally disagree*) to 7 (*totally agree*).

##### Job crafting

Job crafting was measured by the Job Crafting Scale developed by Tims et al. (2012). It is a 21-item scale. To fit the business operation situation, we used a shortened 11-item version. Sample items are, “I try to learn new things in work,” and “I seek advice and guidance from my supervisor.” The scale was measured on a seven-point Likert scale, ranging from 1 (*totally disagree*) to 7 (*totally agree*).

##### Objective performance

Employees’ objective performance was measured at Time 3 using their archival sales performance records for the 1-month period following the Time 2 survey. This sales performance record was the standardized value of sales (i.e., *z*-score) from all insurance products sold by each employee in our sample. We received standardized sales records rather than raw data due to the proprietary and sensitive nature of the latter.

##### Control variables

Employees’ demographics measured at Time 1, including gender, age, and organizational tenure, were included as control variables in the analyses since they could be related to engagement (e.g., de Lange et al., 2008; Van den Broeck et al., 2008) and performance (e.g., Bakker et al., 2012). In sum, by controlling for these individual difference variables, we were able to take possible confounding into account and thus rule out some of the alternative explanations that need to be considered when examining competitive attitude and behavior.

Data collection was facilitated by the HR department of the insurance company. They announced the investigation and sent the link via smartphone within their telecommunity. At Time 1 (the early beginning of a month), participants finished the online survey including trait competitiveness, competitive attitude, and competitive behavior. They also provided their demographic information, including gender, age, and tenure. At Time 2 (the early beginning of next month), employees completed the job crafting and competition climate scales via their smartphone, again with a special link. At Time 3, the end of the second month, we collected the sales performance data (monthly sales revenue) for each employee from the company.

### Results and Discussion

#### Descriptive Analysis

The descriptive statistics, correlations, and reliability coefficients among study variables are reported in Table 3. Gender was significantly related to trait competitiveness (T1) and competitive behavior (T1 and T2), and tenure was significantly related to job crafting and performance. Specifically, trait competitiveness, competitive attitude, and competitive behavior at Time 1 were significantly correlated with each other (*r*s between 0.35 and 0.60, *p* = 0.01). Moreover, competitive attitude and competitive behavior at Time 1 were significantly correlated with job crafting at Time 2 (*r* = 0.21, *p* = 0.00; *r* = 0.21, *p* = 0.00) and performance at Time 3 (*r* = 0.18, *p* = 0.00; *r* = 0.22, *p* = 0.00). Additionally, competitive attitude and competitive behavior were only moderately correlated to each other, providing further evidence that they are empirically distinct from each other. Next, we test our research hypotheses one by one.

**Table 3:** Means, standard deviations, correlations, and reliability estimates in Study 2.

Variable	Mean	*SD*	1	2	3	4	5	6	7	8	9	10	11
(1) Gender (T1)	1.48	0.50	–										
(2) Age (T1)	24.83	4.64	0.22**	–									
(3) Tenure (T1)	12.53	15.22	0.14*	0.29**	–								
(4) Trait competitiveness (T1)	3.94	0.67	-0.19**	0.01	0.05	(0.75)							
(5) Competitive attitude (T1)	3.79	0.82	-0.07	-0.01	0.06	0.35**	(0.87)						
(6) Competitive behavior (T1)	3.82	0.68	-0.20**	0.07	0.04	0.60**	0.35**	(0.83)					
(7) Competitive attitude (T2)	3.80	0.83	-0.06	-0.02	-0.06	0.31**	0.57**	0.32**	(0.91)				
(8) Competitive behavior (T2)	3.80	0.69	-0.23**	0.12*	0.02	0.39**	0.34**	0.47**	0.39***	(0.85)			
(9) Competitive climate (T2)	3.62	0.74	-0.05	0.10	0.21**	0.19**	0.12**	0.23**	0.13**	0.33**	(0.72)		
(10) Job crafting (T2)	4.05	0.56	-0.02	0.03	-0.09	0.16**	0.21**	0.21**	0.33***	0.35**	0.39**	(0.93)	
(11) Performance (T3)	3.15	1.91	0.09	0.17**	0.44**	0.22**	0.18**	0.22**	0.13*	0.11	0.32**	0.10	–

*N = 283. SD, standard deviation. Cronbach’s alpha values are presented on the diagonal, and correlations are presented below the diagonal. Gender: 1 = male, 2 = female. Tenure: It’s measured in months.*

*^∗^*p* < 0.05, ^∗∗^*p* < 0.01, two tailed*.

#### Trait Competitiveness as the Antecedent Variable of Competitive Attitude and Behavior

We used a hierarchical regression to test the effect of trait competitiveness on competitive attitude and competitive behavior. The unstandardized coefficient estimates are reported in Table 4. After controlling for gender, age, and tenure, step 2 showed that competitive attitude (*B* = 0.34, SE = 0.06, *p* = 0.00, 95% CI [0.23, 0.46]) and competitive behavior (*B* = 0.42, SE = 0.05, *p* = 0.00, 95% CI [0.32, 0.51]) were significantly related to trait competitiveness and accounted for additional 8% and 15% of variance, respectively. These results support Hypothesis 1, suggesting that trait competitiveness can be an antecedent of competitive attitude and behavior.

**Table 4:** Regression analyses for competitive attitude (T2) and competitive behavior (T2).

Variables	Competitive attitude (T2)				Competitive behavior (T2)			
	Δ*R*^2^	*B*	*SE*	95% CI	Δ*R*^2^	*B*	*SE*	95% CI
Step 1:	0.01				0.05^∗∗^			
Intercept		-0.08^†^	0.04	[-0.16, 0.00]		-0.06^†^	0.03	[-0.12, 0.01]
Gender		-0.05	0.08	[-0.21, 0.12]		-0.20**	0.07	[-0.34, -0.06]
Age		0.00	0.01	[-0.02, 0.02]		0.02*	0.01	[0.00, 0.03]
Tenure		0.00	0.00	[-0.01, 0.01]		0.00	0.00	[-0.00, 0.01]
Step 2:	0.08^∗∗^				0.15^∗∗^			
Trait competitiveness		0.34**	0.06	[0.23, 0.46]		0.42**	0.05	[0.32, 0.51]
								
*R*^2^		0.08				0.19		
*F*		9.26**				25.05**		

*N = 431. SE, standard error. *B*, unstandardized coefficient. 95% CI, 95% confidence interval. ^†^*p* < 0.10, ^∗^*p* < 0.05, ^∗∗^*p* < 0.01, two tailed*.

#### The Dual-Model of Competitive Attitude and Competitive Behavior for Performance and Job Crafting

**FIGURE 2:**
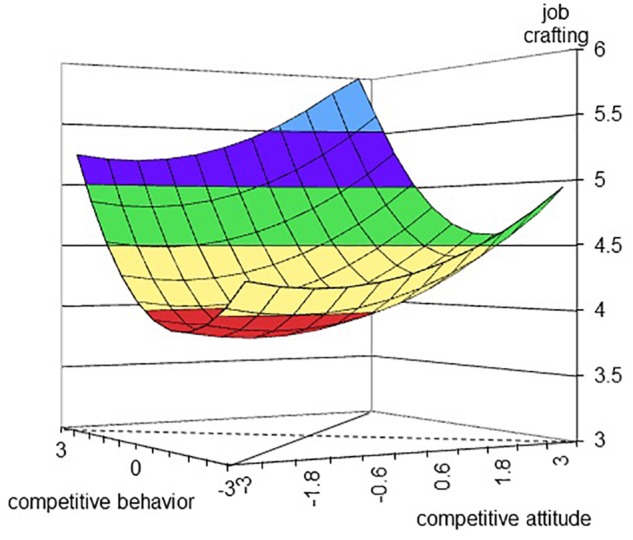
Effect of competitive behavior and competitive attitude on job crafting. The dashed line represents the incongruent line, along with competitive behavior becomes lower and competitive attitude becomes higher from the left corner to the right corner. The full line represents the congruent line, along with competitive behavior and attitude becomes higher from the front corner to the rear corner.

Next, we performed polynomial regressions (Edwards, 1994; Edwards and Cable, 2009) to examine the dual-model. Hypothesis 2a predicted a congruence effect of competitive attitude and behavior on job crafting. The first column in Table 5 displays the estimated coefficients as well as the slopes and curvatures along the congruence line and incongruence line for job crafting. The response surface is plotted in Figure 2. The last row in Table 5 shows that the three second-order polynomial terms are marginally significant (*F* = 2.29, *p* = 0.08). The incongruence line curved upward (curvature = 0.12, *p* = 0.12). This suggested that the incongruence line was U-shaped, and that the lowest point of job crafting was not the extremity. That is, any deviation from the congruence line did not decrease job crafting. Therefore, Hypothesis 2a was not supported.

**Table 5:** Polynomial regressions of job crafting and performance on competitive attitude and behavior congruence/incongruence.

Variables	Job crafting			Performance		
	*B*	*SE*	95% CI	*B*	*SE*	95% CI
**Step 1:**						
Intercept	4.00**	0.04	[3.92, 4.08]	3.16**	0.13	[2.91, 3.41]
Gender	0.04	0.07	[-0.09, 0.18]	0.25	0.21	[-0.16, 0.67]
Age	0.01	0.01	[-0.01, 0.02]	0.01	0.02	[-0.03, 0.06]
Tenure	-0.01*	0.00	[-0.01, -0.00]	0.05**	0.01	[0.04, 0.07]
**Step 2:**						
Competitive attitude (CA)	0.12*	0.05	[0.03, 0.22]	0.25	0.15	[-0.05, 0.56]
Competitive behavior (CB)	0.18**	0.06	[0.06, 0.30]	0.48*	0.19	[0.11, 0.85]
CA × CB	0.00	0.05	[-0.10, 0.10]	0.01	0.16	[-0.30, 0.32]
CA^2^	0.03	0.04	[-0.04, 0.11]	0.06	0.12	[-0.18, 0.29]
CB^2^	0.09^†^	0.05	[0.00, 0.18]	-0.05	0.14	[-0.34, 0.23]
*R*^2^	0.10			0.25		
Congruence (CA = CB) line						
Slope	0.30**	0.06		0.73**	0.18	
Curvature	0.13**	0.05		0.02	0.19	
Incongruence (CA = -CB) line		.				
Slope	-0.05	0.09		-0.23	0.29	
Curvature	0.12	0.08		-0.01	0.25	
*F* for the three quadratic terms	2.29^†^			0.11		

*N = 283. Unstandardized regression coefficients are reported. SE, standard error. ^†^*p* < 0.10, ^∗^*p* < 0.05, ^∗∗^*p* < 0.01, two tailed*.

Hypothesis 2c suggested that job crafting would be higher when both the levels of competitive attitude and behavior are high. The results of regression showed that, after controlling for gender, age, and tenure, competitive attitude (*B* = 0.11, SE = 0.04, *p* = 0.01, 95% CI [0.03, 0.20]), competitive behavior (*B* = 0.13, SE = 0.05, *p* = 0.01, 95% CI [0.03, 0.24]), and their interaction term (*B* = 0.04, SE = 0.05, *p* = 0.46, 95% CI [-0.06, 0.13]) were positively related to job crafting. But the coefficient of interaction term was not significant. Therefore, Hypothesis 2c was partially supported.

**FIGURE 3:**
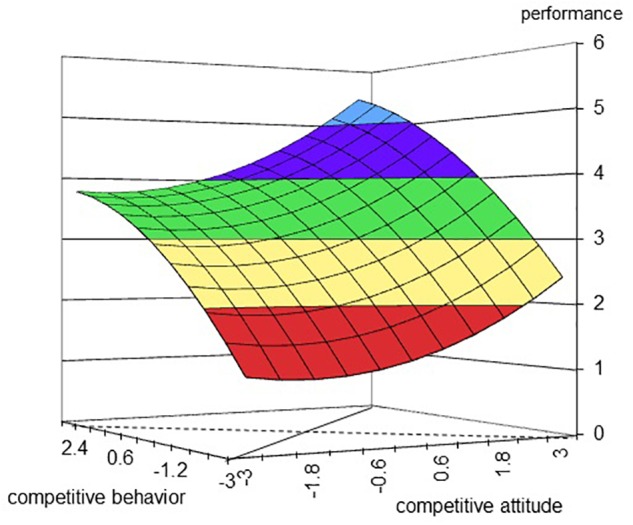
Effect of competitive behavior and competitive attitude on performance. The dashed line represents the incongruent line, along with competitive behavior becomes lower and competitive attitude becomes higher from the left corner to the right corner. The full line represents the congruent line, along with competitive behavior and attitude becomes higher from the front corner to the rear corner.

Hypothesis 2b predicted that performance is higher when competitive attitude and behavior are aligned at a high level compared to a low level. The second column in Table 5 shows a negative slope (slope = -0.23, *p* = 0.44) along the incongruence line when the curvature is negative (curvature = -0.01, ns), meaning the line was inversely U-shaped (also see Figure 3). That is, any deviation from the congruence line did decrease performance. Hypothesis 2b was supported.

Hypothesis 2d suggested that performance would be higher when the levels of competitive attitude and behavior are high. The results of regression showed the coefficients of competitive attitude (*B* = 0.21, SE = 0.13, *p* = 0.11, 95% CI [-0.05, 0.47]), competitive behavior (*B* = 0.53, SE = 0.16, *p* = 0.00, 95% CI [0.21, 0.85]), and their interaction term (*B* = 0.03, SE = 0.15, *p* = 0.84, 95% CI [-0.26, 0.32]) were positive. But the interaction term was not significant. Therefore, Hypothesis 2d was partially supported.

#### The Mediating Effect of Job Crafting and the Moderating Effect of Competitive Climate

We conducted hierarchical regression analyses to examine the moderated mediating effects with PROCESS in SPSS (22.0.0.0), and the results are presented in Table 6. After controlling for gender, age, and tenure, competitive attitude (T1) was significantly and positively correlated with performance (*B* = 0.36, SE = 0.12, *p* = 0.00, 95% CI [0.11, 0.60]). In Model 3, job crafting was positively but marginally correlated with performance (*B* = 0.36, SE = 0.19, *p* = 0.05, 95% CI [-0.00, 0.73]) when competitive behavior (T1) was entered into the regression analysis. The mediating effect of job crafting on the relationship between competitive behavior and performance was significant (indirect effect = 0.05, 95% bias-corrected CI [0.00, 0.14]). Hypothesis 3a was supported.

**Table 6:** Hierarchical regression results (Study 2).

Variables	Path 1			Path 2		
	Performance	Job crafting	Performance	Performance	Job crafting	Performance
	Model 1 *B*(*SE*)	Model 2 B(SE)	Model 3 *B*(*SE*)	Model 4 *B*(*SE*)	Model 5 *B*(*SE*)	Model 6 *B*(*SE*)
Intercept	3.16^∗∗^(0.10)	-0.01(0.03)	3.17^∗∗^ (0.10)	3.17^∗∗^ (0.10)	-0.02 (0.03)	3.17^∗∗^ (0.10)
Gender	0.11 (0.21)	0.03 (0.06)	0.11^∗∗^ (0.21)	0.25 (0.21)	0.05 (0.06)	0.24 (0.21)
Age	0.02 (0.02)	0.01 (0.01)	0.01 (0.02)	0.01 (0.02)	0.00 (0.01)	0.00 (0.02)
Tenure	0.05^∗∗^ (0.01)	-0.01^∗∗^ (0.00)	0.05^∗∗^ (0.01)	0.05^∗∗^ (0.01)	-0.01^∗∗^ (0.00)	0.05^∗∗^ (0.01)
Path 1						
Competitive attitude (T1)	0.36^∗∗^(0.12)	0.11^∗∗^ (0.04)	0.30^∗^ (0.13)			
Competitive climate		0.30^∗∗^ (0.04)				
CA × CC		0.09^∗^ (0.04)				
Job crafting			0.36^†^ (0.19)			
Path 2						
Competitive behavior (T1)				0.61^∗∗^ (0.15)	0.13^∗∗^ (0.05)	0.56^∗∗^ (0.15)
Competitive climate					0.29^∗∗^ (0.04)	
CB × CC					0.16^∗∗^ (0.05)	
Job crafting						0.32^†^ (0.18)
*R*^2^	0.22	0.22	0.23	0.24	0.22	0.25
*F*	19.59^∗∗^	13.35^∗∗^	16.59^∗∗^	22.14^∗∗^	13.26^∗∗^	18.46

*N = 283. *B*, unstandardized coefficient; SE, standard error. Gender: 1 = male, 2 = female. Tenure: It is measured in months. CA, competitive attitude; CB, competitive behavior; CC, competitive climate; CA × CC, the interaction term of CA and CC; CB × CC, the interaction of CB and CC. Path 1: competitive attitude → job crafting → performance. Path 2: competitive behavior → job crafting → performance. ^†^*p* < 0.10, ^∗^*p* < 0.05, ^∗∗^*p* < 0.01, two tailed*.

**FIGURE 4:**
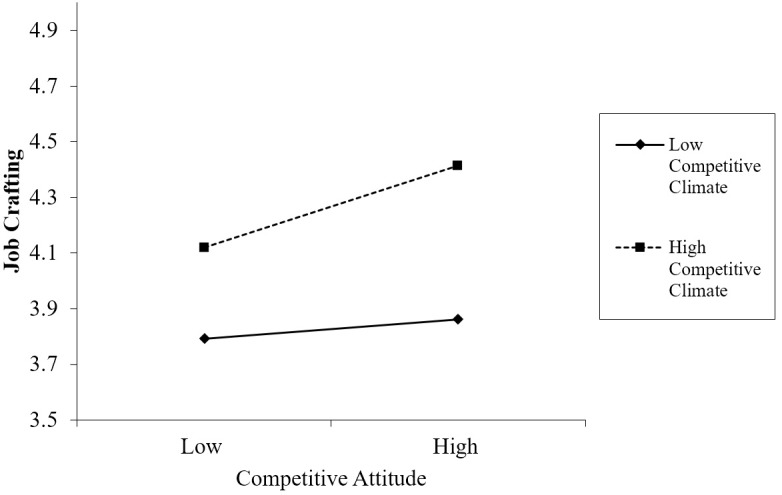
The moderating effect of competitive climate on the relationship between competitive attitude and job crafting.

We tested the moderating effect of competitive climate in Model 2; the result showed that the interaction between competitive attitude and competitive climate was significant (*B* = 0.09, SE = 0.04, *p* = 0.03, 95% CI [0.01, 0.18]). The simple slope test showed that when the competitive climate was high (Mean + SD), the relationship between job crafting and competitive attitude was stronger (*B* = 0.18, *p* = 0.00) than when the competitive climate was low (*B* = 0.04, *p* = 0.36; Mean - SD) (the interaction is plotted in Figure 4). Hypothesis 4a was supported. Additionally, the indirect effect of competitive attitude on performance via job crafting was significant and positive (conditional indirect effect = 0.18, 95% bias-corrected CI [0.08, 0.27]) when the competitive climate was high. The indirect effect became weaker (conditional indirect effect = 0.04, 95% Bias-corrected CI [-0.06, 0.14]) in a low competitive climate. Thus, Hypothesis 5a was supported.

Similarly, competitive behavior (T1) was significantly and positively correlated with performance (*B* = 0.61, SE = 0.15, *p* = 0.00, 95% CI [0.32, 0.91]). Additionally, job crafting (T2) was positively correlated with performance (*B* = 0.32, SE = 0.18, *p* = 0.08, 95% CI [-0.04, 0.68]) when competitive behavior (T1) was entered into the regression. The mediating effect of job crafting on the relationship between competitive behavior and performance was significant (indirect effect = 0.06, 95% Bias-corrected CI [0.00, 0.16]). Hypothesis 3b was supported.

**FIGURE 5:**
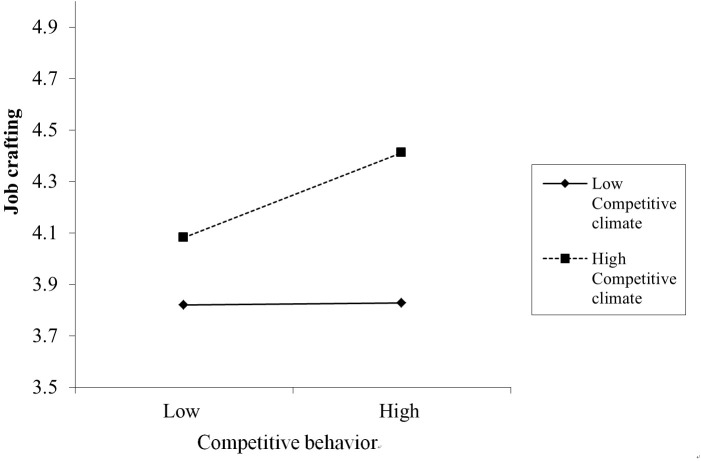
The moderating effect of competitive climate on the relationship between competitive behavior and job crafting.

Moreover, the moderating effect of the competitive climate on the relationship between competitive behavior and job crafting in step 2 was significant (*B* = 0.16, *SE* = 0.05, *p* = 0.00, 95% CI [0.06, 0.27]). The simple slope test showed that when the competitive climate was high, the relationship between job crafting and competitive behavior was stronger (*B* = 0.24, *p* = 0.00) than when the competitive climate was low (*B* = 0.01, *p* = 0.93). The interaction plot is presented in Figure 5. Hypothesis 4b was supported. The results also showed that when the competitive climate was high, the indirect effect of competitive behavior on performance via job crafting was significant and positive (conditional indirect effect = 0.08, 95% bias-corrected CI [0.01, 0.19]). Whereas when the competitive climate was low, the indirect effect was weaker (conditional indirect effect = 0.00, 95% bias-corrected CI [-0.06, 0.08]). Thus, Hypothesis 5b was supported.

## General Discussion

### Main Findings

The current research developed two new concepts, competitive attitude and competitive behavior, to show that they can interact with competitive climate to influence job crafting and job performance, as they are more proximal to job behavior. We found that trait competitiveness can be an antecedent of competitive attitude and competitive behavior. Competitive attitude and competitive behavior can relate to job performance via being associated with job crafting, and this relationship will be moderated by environmental competitive climate. In particular, competitive attitude and competitive behavior simultaneously influence job behavior in different ways.

### Theoretical Contribution

Our research contributes to the literature in several ways. First, we contribute to the literature on competition at the individual level. Competition is a prevalent phenomenon (Thiel, 2017) and is viewed a component of basic human nature (Churchill et al., 1997; Klein and Miller, 1998; Matsumoto and Willingham, 2006; Dohmen et al., 2011). The existing research mainly focuses on static factors, such as trait competitiveness (Spence and Helmreich, 1983). We proposed two dynamic factors including competitive attitude and competitive behavior to show that competition behavior can be driven by some internal psychological characteristics that can be changeable and adaptable under different environments. This can contribute to the understanding of the mechanisms and ways to increase competitive ability. Particularly, we developed two new psychological measures, one for competitive attitude and one for competitive behavior, and provided preliminary evidence for their reliability and validity. They can be useful tools for further research and practice.

Second, we contribute to the literature on self-determination theory (Deci, 1980; Ryan and Deci, 2000, 2006, 2017). According to the self-determination theory, human beings have some internal psychological strength that drives behavior to pursue goals. Competitive attitude and competitive behavior can be two internal factors that help individuals determine their behaviors, cope with competition and outperform others. In particular, we showed that competitive attitude and competitive behavior work separately and simultaneously to influence job behavior (job crafting) and results (job performance). The results suggest that competitive attitude and behavior are different from the trait competitiveness. They are dynamic and can be adaptable to determine employees’ behaviors, whether trait competitiveness is high or low.

Third, our research contributes to the literature on person–environment fit theory in the field of competition. The person–environment (P–E) fit perspective claims that people actively interact with the environment and adjust behaviors to adapt to the environment (Endler and Magnusson, 1976; Pervin, 1989; Edwards, 1991; Kristof, 1996; Kristof-Brown et al., 2005). Consistent with this claim, our results demonstrate that competitive attitude/behavior interact with competitive climate to influence employees’ working behavior and outcomes. It suggests that both employee (the Person) and the organization (the Environment) are agents that affect working behavior and performance.

Fourth, our findings explain the path that links trait competitiveness, competitive attitude and behavior, job crafting, and the job performance. In particular, our findings demonstrate that job crafting (Wrzesniewski and Dutton, 2001; Berg et al., 2008; Parker and Ohly, 2008) can explain the mechanism how competitive attitude and competitive behavior relate to job performance. This link helps understand the connections among trait—attitude—behavior—outcomes (performance) involved in competition.

### Limitations and Future Research

The present research also has some limitations that need to be addressed. First, although we have obtained some preliminary evidence for the reliability and validity of the two new measures of competitive attitude and competitive behavior, their usefulness across multiple samples still needs to be examined. This is particularly important because they are both dynamic psychological variables that may change from time to time.

Second, this research was conducted in an insurance company. The insurance industry is quite different from other industries because of the fierce competition. Whether or not the findings can be replicated in other industries needs to be established. However, we argue that because of the feature of fierce competition in insurance industry, the findings from this industry are more salient. Further research should test the generality of the findings from the current research.

Third, the differentiation of the trait competitiveness from competitive attitude and competitive behavior should be further tested in future studies. More evidence needs to be obtained to demonstrate the distinctions between trait-like variable measure and dynamic variable measures.

Fourth, both studies in the current research use correlational methods. Although the second study used a time-lagged design with multiwave data, the causality relationship among the tested variables remains unclear. Further research should address this issue by using experimental method or other causality research design.

Finally, we note that our Hypothesis 2a was not supported. This hypothesis predicted a congruence effect of competitive attitude and behavior on job crafting. However, the results suggested that the congruence and incongruence line were U-shaped. That is, when both competitive attitude and behavior were either high or low simultaneously, job crafting was moderate, whereas when both competitive attitude and behavior were at the middle level, job crafting was the lowest. One possible explanation is that there are some other factors that influence job crafting, and job crafting only partially mediated the relationship of competitive attitude and behavior with job performance. Future research should test why this happens. Nevertheless, some points are still clear: (1) when both competitive attitude and behavior are high, job crafting reaches the highest level, and (2) when one of the two components – competitive attitude and competitive behavior – is high, job crafting is still quite high. In addition, Hypothesis 2c and 2d were only partially supported. The results just showed a promoting tendency of competitive attitude and competitive behavior to job crafting (or performance), but the interaction coefficients of regression were not significant. The alternative explanation was that competitive attitude and competitive behavior might work out separately and directly on job crafting and performance, according to our dual-component model. Future research should incorporate more efforts to clarify whether competitive attitude and competitive behavior take effect independently or interactively.

### Implications for Practice

The findings from the present research can be useful in practice in many ways. For example, organizations can recruit those people high in competitive attitude and competitive behavior, though they may be low in trait competitiveness, in an effort to seek high performance. Moreover, organizations can strengthen their competitive climate to serve as a powerful environmental force (Kohn, 1992; Brown et al., 1998) to drive employees’ competitive attitude and competitive behavior. By doing so, employees will show more job crafting behavior to obtain better performance (Berg et al., 2010).

As for individuals, it is true that not everyone has a high level of trait competitiveness. Does it mean these people will definitively lose in a competition? Fortunately, according to the P–E fit perspective (Endler and Magnusson, 1976; Pervin, 1989; Edwards, 1991; Kristof, 1996; Kristof-Brown et al., 2005) and the findings in our study, those who are low in trait competitiveness can also learn in the competitive climate to adjust their competitive attitude and behavior to fit the job demands, increase their job crafting and thus outperform others. It is because of that capacity that people can also have dynamic psychological characters that are different from static-like dispositions such as trait competitiveness. Competitive attitude and competitive behavior are the two dynamic features that people can learn to embody that allow them to compete with others. People will interact with the organization, given that they are influenced by the environment, to learn how to adjust their attitude and behavior to fit the competition demands. Organizations can also provide training to help employees increase their competitive attitude and behavior. By doing so, employees will show more job crafting behavior and job performance that will ultimately benefit both employees and the organization.

## Author Contributions

HW and LW proposed the research idea and made the research design. HW, LW, and CL collected the data and prepared the manuscript. CL and LW ran the data analysis.

## Conflict of Interest Statement

The authors declare that the research was conducted in the absence of any commercial or financial relationships that could be construed as a potential conflict of interest.
