# Supplementary material for: Employee Competitive Attitude and Competitive Behavior Promote Job-Crafting and Performance: A Two-Component Dynamic Model
Source: Front Psychol. 2018 Nov 21;9:2223. doi: 10.3389/fpsyg.2018.02223 (PMC6258773; doi:10.3389/fpsyg.2018.02223)
Supplement: Supplementary file 1 [file Data_Sheet_1.docx]

**Appendix:**

**Competitive attitude scale**

1. I hate competition.
2. I feel competition very tiresome.
3. Competition makes me feel disgust.
4. I think competition will destroy interpersonal harmony and cooperation.

**Competitive behavior scale**

1. I try to be the best in the team.
2. I put effort to win out.
3. I take my best to surpass any others.
4. I always attempt to do better than others.
5. I strive for first place.
